# Recovery of Industrial Wastes as Fillers in the Epoxy Thermosets for Building Application

**DOI:** 10.3390/ma14133490

**Published:** 2021-06-23

**Authors:** Jakub Hodul, Lenka Mészárosová, Rostislav Drochytka

**Affiliations:** Department of Technology of Building Materials and Components, Faculty of Civil Engineering, Brno University of Technology, Veveří 331/95, 602 00 Brno, Czech Republic; meszarosova.l@fce.vutbr.cz (L.M.); drochytka.r@fce.vutbr.cz (R.D.)

**Keywords:** epoxy resin, filler, composite, adhesion, chemical resistance, microstructure, strength

## Abstract

Epoxy resins are currently used in many areas of construction, such as resistant coatings, anchors, fibre-reinforced polymer (FRP) composites, grouts, etc. This paper deals mainly with epoxy composites that can be applied during the rehabilitation of concrete constructions. The influence of a filler type on epoxy thermoset composites was monitored, whilst three different types of epoxy resin were used in order to achieve a better representation and confirmation of the results. During the testing of fillers, these were mainly secondary raw materials, including pre-treated hazardous waste (neutralisation sludge), representing various shapes and sizes of particle, while their amount in the epoxy matrix was chosen with regard to optimal viscosity and workability. Physical and mechanical parameters, like compressive and flexural strengths, cohesion with the concrete and thermal expansion of the epoxy composites containing various fillers were determined. The microstructure of epoxy composites with a different filler type and chemical resistance against chemical aggressive media were all monitored. The microstructure of epoxy composites was monitored using scanning electron microscopy (SEM) supported by energy-dispersive X-ray spectroscopy (EDX). Computed tomography (CT) was also used for the evaluation of the cohesion of the epoxy composites with concrete and dispersion of the filler in the epoxy matrix.

## 1. Introduction

Polymer composites are often used as binding agents of rehabilitation materials of building constructions [1,2]. Epoxy resins are frequently used as coatings, adhesives, high-performance composite materials [3,4,5] and spattle coats [6].

Epoxy resins are polymeric substances that are mainly formed by cross-linking when an epoxy class enters the reaction [7].

During the curing of epoxide resins, crosslinking between the epoxy molecules and reactive groups on each end of the curing agent occurs [8].

**Figure 1:**
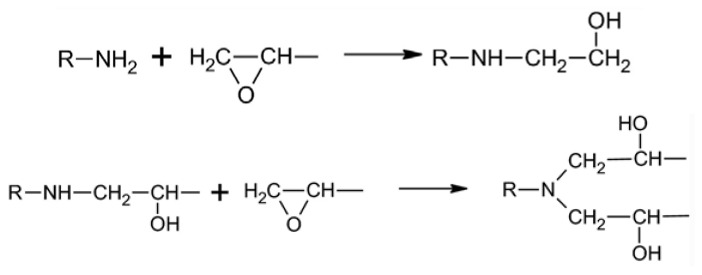
The polyaddition reaction of amines (the amine reacts with the epoxide oxygen and forms a hydroxyl group) [9].

The principle of epoxy resins curing using polyamines is stated in Figure 1.

The means of crosslinking fundamentally affects the properties of epoxy thermosets. Generally, epoxy resins have excellent cohesion with a substrate, low shrinkage and good permeability resistance to water, acids, alkalis and other corrosive substances [6].

The monitoring of the compressive strength of polymeric concrete and polymeric mortars based on epoxy resins has already been described in many previous scientific works. It was observed that the compressive strength depends mainly on the resin content [10]. Rebeiz et al. [11], proved that by adding 15% of fly ash into the resin, the compressive strength increases by up to 30%. The application of the filler significantly influences the change in the mechanical, thermal and processing properties of the epoxy composites [12]. Atzeni et al. [13] dealt with the substitution of a conventional quartz flour filler by using fly ash in epoxy composites based on bisphenol A, and it was found that the results of mechanical properties of the epoxy composites did not differ significantly. Lin et al. [14], examined different approaches to the thermal conductivity of powder-filled epoxy resins depending on various shapes and sizes of particles. A comparative study of the performances of fly ash with epoxy resin reported that a combination of fly ash and epoxy resin can provide higher mechanical strength than epoxy composite containing silica fume. The addition of a filler can improve a matrix’s compressive and tensile properties although its flexural strength may decrease [15]. The mechanical properties of epoxy polymer concrete are influenced by the matrix-to-aggregate ratio and the tensile and flexural properties are dependent on resin content in the epoxy concrete [16]. From an economical point of view, it is recommended that the minimum amount of resin is used to minimise the cost [17], and, therefore, it is important to optimise the mix proportions. Replacing primary raw materials with secondary ones will further improve the economic demands of epoxy composites. By using some suitable by-products, it would even be possible to improve some properties of epoxy composites, not only the physical and mechanical parameters, but also their long-term durability. However, the effect on the properties of a polymer matrix due to an introduction of filler is still unknown [18]. Mainly for these reasons, the dependences of the types of fillers used on the characteristic properties of epoxy composites, such as compressive and flexural strength, adhesion to concrete, but also chemical resistance, are monitored and evaluated in this paper.

For the selection of suitable fillers for epoxy composites, the maximum possible filler content in the polymer matrix and particle size and shape index are important. [19]. In the study by Jin et al. [20], experiments were carried out with the filler ratio (nano-Al_2_O_3_ and nano-SiC particles) within a range of 5–15 wt.% and monitored thermal properties, also focusing on morphology, and use of mineral fillers in the epoxide matrix. The coefficient of linear thermal expansion is important to analyse the processes of structure formation and the behaviour of epoxy composites, with different amounts and types of fillers, under the influence of a thermal field. [21].

It was found that the addition of waste glass powder at an amount of 7.4 to 35.9% has a positive effect on the pull-off strength of epoxy polymer mortars [22]. An irregular shape of the crumble filler significantly increases the strength of epoxy composite compared to the spherical shape of glass powder [23]. It was proven that the tensile and compressive strength of epoxy composites was improved with the increase of fly ash content [24]. Environmental acceptance of waste foundry sands in polymer concrete requires reliable knowledge of the sand composition. The incorporation of waste foundry sands in epoxy composites can contribute to sustainable industrial growth and the production of high-quality polymer concrete [25,26]. Neutralization sludge (NS) is hazardous waste that cannot be used without its complete incorporation into other material, as there is a risk of hazardous pollutants being released into the environment [27]. Heavy metals in neutralization sludge could have a positive effect on the properties of epoxy-based materials [28].

However, a detailed comparison of the dependence of the influence of the filler component’s particle shape on the behaviour of the polymeric compound in terms of rheologic properties and properties of the resulting product was not summarised in any available literature. Following previous research of epoxy composites containing fine waste from the production of mineral wool board insulation [29], and the use of secondary raw materials as fillers in epoxy polymer concrete [30], in which the temperature and chemical resistance of repair composite [31] was also monitored, this paper also studies the influence of the filler particle shape and size based on industrial waste and the properties of the epoxy composite.

By using waste products as fillers in epoxy composites, it is possible to save primary resources and achieve the required properties of epoxy composites in a much more environmentally friendly way. The use of pre-treated hazardous waste could, in particular, reduce the volume of hazardous waste in landfills, as tons of unused waste are landfilled worldwide.

## 2. Materials

### 2.1. Tested Formulations

**Figure 2:**
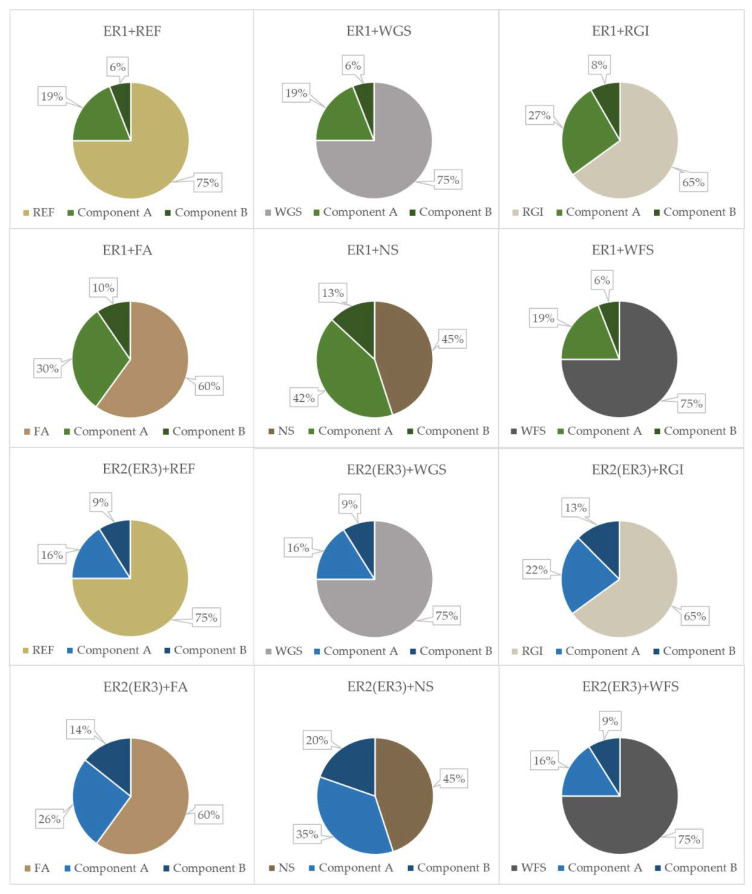
Tested formulations of the epoxy composites (ER—Epoxy resin, REF—Reference filler, WGS—Waste glass from solar panels, RGI—Waste from the production of mineral insulation boards, FA—Fly ash, NS—Neutralisation sludge, WFS—Waste foundry sand).

Proportions between resin (epoxy resin (A), hardener (B)) and fillers are stated in Figure 2. The mix ratio by weight of resin to hardener with ER2 and ER3 binders was the same. Tested materials (epoxy composites) were prepared by first mixing the filler into component A (epoxy resin), mixing slowly for 5 min, then adding component B (hardener), mixing the mixture slowly for 5 min again so that no air was introduced into the mixture. Finally, samples for individual tests were prepared. For the preparation of samples for compressive and flexural strength determination, the fresh mixture was poured into a silicone triple mould sprayed with the demoulding agent and finally tapped lightly with the mould to remove excess air. The silicone mould was also used to prepare the sample for abrasion resistance. After 24 h, the samples were demoulded and conditioned at 23 °C and relative humidity 50% (laboratory conditions) before testing. To prepare samples for the determination of hardness and impact resistance, fresh material was applied onto the surface of the cement-bonded particleboard.

### 2.2. Epoxy Resin

During the experimental verification stage, four types of epoxy resins were used, differing mainly in the type of hardener used—see Table 1. The manufacturer Lena Chemical, Ltd. (Sternberk, Czech Republic) supplied the epoxy resin and hardener for the ER1 binder, and IN-CHEMIE Technology, Ltd. (Olomouc, Czech Republic) supplied the epoxy resins and hardeners for ER2 and ER3 binders. Properties of the epoxy binders are stated in Table 2. To verify the influence of the type of filler on the physical and mechanical parameters of epoxy composites, it is more appropriate to use a number of different types of epoxy resins in order to improve reproducibility and confirm the results. All epoxy resins used can be characterised by Bfl (combustible materials—very limited contribution to fire floorings)-S1 (quantity/speed of smoke emission during combustion absent or weak) classification for reaction to fire according to the standard EN 13501-1 [32].

### 2.3. Fillers

Based on the differences in the morphology of the grain, the fillers were chosen using the widest spectrum of possible shape properties. Another evaluation criterion for the selection of input raw materials was the maximum possible use of secondary raw materials and waste materials from which a significant reduction of the ecological impacts of industrial production can be obtained.

#### 2.3.1. Reference Filler—Quartz Sand (REF)

The quartz sand mixtures (Chejn, Ltd., Sušice, Czech Republic) were used as the reference filler. They are impurity-free and have the optimal round grain shape mainly used as a filler for polymer concrete (PC), resins, grouts, grouting and backfills. The sand fraction of 0–1.5 mm (ISG A1) was used within the research, which is commonly used for epoxy mortars.

#### 2.3.2. Waste Glass from Solar Panels (WGS)

The solar (photovoltaic—PV) panels, that were used in this work, are manufactured by the QS Solar company (Nantong) in China. These panels are thin-layer modules with a base made of amorphous SiO_2_ and have not been polluted by other materials. Currently, it is estimated that the service life of the panels, defined by a 20% reduction in efficiency, will remain at a sufficient quality level for 30–40 years following installation. However, in most cases, the main reason for the disposal of a panel is due to mechanical damage caused during transportation or installation. The biggest problem for lower-quality panels is delamination, whereby the ‘sandwich’ structure of a PV panel comes apart due to the effects of temperature and UV radiation. In order to recycle PV panels, the PV Cycle system was devised. This is a Europe-wide activity engaged in by the manufacturers and importers of photovoltaic (PV) panels based on voluntary responsibility for the product during its whole service life. One of the containers is intended for crystal quartz panels, and the second container is intended for thin-layer panels, for which different recycling is used. Glass accounts for the largest part of the weight of crystal PV panels (60–70%) and the aluminium frame (approximately 20%), while for thin-layer panels the glass and aluminium parts account for at least 95%. During 2010, the highest number of panels were installed in the Czech Republic, namely 160,000 tons of PV panels, for which their service life is estimated to end in 2040 [33]. When using supplied, already non-functioning panels, the aluminium frames had to be removed first. The top glass layer was exceedingly difficult to remove from the polymeric surface, and that is why the panel had to be thrown into the ball mill OM(tumbling drum)-20f (BRIO Hranice, Ltd., Hranice, Czech Republic) for approximately 20 min following the removal of all metal parts, where the glass became separated from its tenacious polymeric underlay. Individual parts were then ground using the ball mill down to the required size (fraction of 0–1.5 mm) for approximately ten minutes.

#### 2.3.3. Waste from the Production of Mineral Insulation Boards (RGI)

The waste from the production of insulation boards made of mineral wool and containing a high proportion of recycled glass (>80%) was selected as another perfect filler in the form of a secondary raw material for the rehabilitation masses being developed. This is the dry by-product of the production process that falls off from below a pulper in front of a hardening chamber so it does not contain any organic elements, and it is, therefore, possible to classify this as recycled glass without organics.

#### 2.3.4. Fly Ash (FA)

The filter fly ash from the thermal power plant (Veolia, Plc., Třebovice, Czech Republic) from the combustion of hard coal was also selected as a filler. FA was contaminated by the influence of the flue gas denitrification (DeNOx) using selective, Selective Non-Catalytic Reduction (SNCR), when a urea solution (CO(NH₂)₂) is injected into a boiler at high temperature.

#### 2.3.5. Neutralisation Sludge (NS)

The neutralisation sludge is created as a by-product during the surface treatment of metal elements (ŽDB, Plc., Bohumín, Czech Republic). The objective of galvanic plating is to create a metal coating on predominantly metallic base materials. The protective, anticorrosive layer shields the product against the influences of the environment, thus, extending their useful service life. During the galvanic plating process, a number of waste products are generated—sludges and filter cakes from the neutralisation station—that contain dangerous substances. According to the European Waste Catalogue (EWC), the selected hazardous waste (HW) is classified under code 19 02 05—Sludges from physical and chemical treatment containing hazardous substances. These types of sludge are characterised by several dangerous properties such as HP5 (Specific Target Organ Toxicity/Aspiration Toxicity), HP14 (Ecotoxic) and HP15 (Waste capable of exhibiting a hazardous property listed in Annex III of the 2008/98/ES Directive, not directly displayed by the original waste). This sludge had to be dried and ground by the vibratory disc mill RS 200 (Retsch GmbH, Haan, Germany) down to a suitable granulometry, which would represent finer fillers (FA, RGI) and so that it can be successfully uniformly dispersed in the polymer matrix, prior to usage itself.

#### 2.3.6. Waste Foundry Sand (WFS)

In the Czech Republic, the annual consumption of foundry sands is about 800,000 tons, of which only less than 10% is recycled. Bentonite or cement-bonded sands and water glass mixtures are practically environmentally friendly. Due to their variable nature, natural foundry sands are being increasingly replaced by synthetic sands, into which the specified amount of bonding admixture is added (bentonite on most occasions) [34]. The sustainable usage of WFS provides an economical and environmentally friendly solution compared to the high costs of disposal in landfills and extraction of primary raw materials [35]. The used WFS was adjusted to a granulometry of less than one millimetre in a foundry, so it does not need to be pre-treated before further use.

#### 2.3.7. Summary of Properties of Input Raw Materials

**Figure 3:**
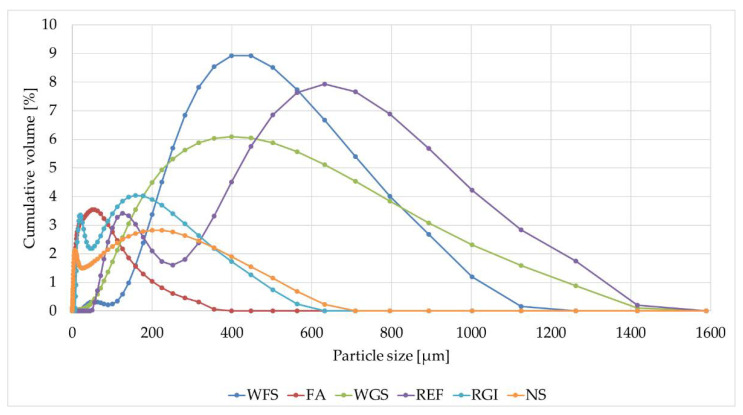
Particle size distribution of the fillers determined by Mastersizer 2000 laser diffraction particle size analyser (REF—Reference filler, WGS—Waste glass from solar panels, RGI—Waste from the production of mineral insulation boards, FA—Fly ash, NS—Neutralization sludge, WFS—Waste foundry sand).

**Figure 4:**
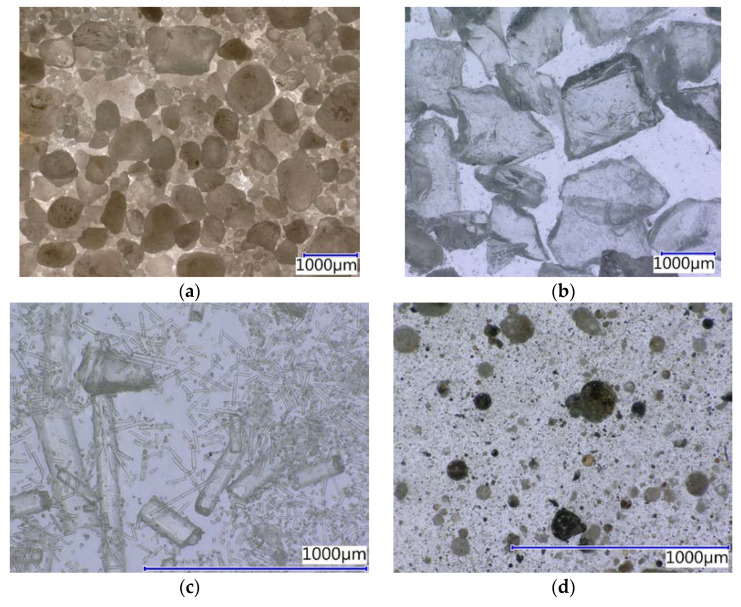

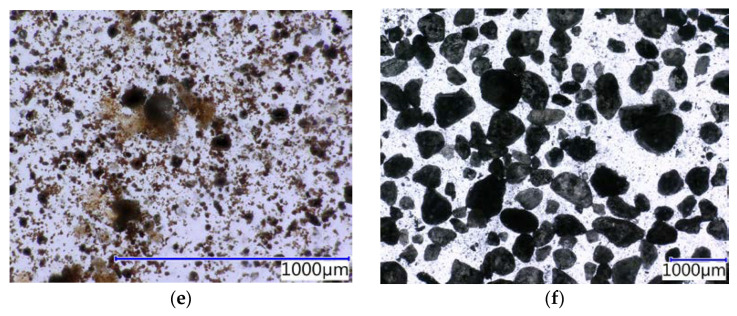
The structure of individual filler types: (**a**) REF—Silica sand (16×); (**b**) WGS—Waste glass from the QS solar panels (16×); (**c**) RGI—Recycle from the production of mineral glass insulation (67×); (**d**) FA—Fly ash (67×); (**e**) NS—Neutralisation sludge (67×); (**f**) WFS—Waste foundry sand (16×).

The fillers were characterised by the determination of chemical composition (Table 3, particle size distribution (Figure 3), density and specific surface area (Table 4) and particle shape (Figure 4).

As can be seen from the size distribution curve (Figure 3), the types of filler used showed fine-grained particles with different sized grains. Three filler types (FA, RGI, NS) were selected with a particle size less than 0.63 mm (fine-grained) and three filler types (REF, WFS, WGS) were selected with a particle size less than 1.6 mm (coarse-grained). These fractions were chosen to monitor the effect of filler particle size on the tested properties of epoxy composites.

To study the stage of synergic influence of a filler and epoxy resins, fillers were used, based on the different particle shapes (in Figure 4, it is possible to see the characteristic grain shape of individual fillers) from spherical, to arched sharp-edged, to acicular, among others.

## 3. Methods

### 3.1. Compressive and Flexural Strength

Compressive and three-point flexural strength was determined pursuant to the EN 12808-3 [36] on specimens shaped like small beans with dimensions of 20 mm × 20 mm × 100 mm seven days after specimen preparation. This standard allows samples of these dimensions to be used, and it is possible to subsequently determine the compressive strength using the fractions of beams. There are chemically resistant epoxy-based grouts on the market with quartz sand as filler, which are basically also epoxy composites, so it is possible to use this standard. The distance between the supports was 80 mm and the pressure area during compressive loading was 400 mm^2^. The testing pressure RT 200/10-1 D servo (ratioTEC Prüfsysteme GmbH, Langenenslingen, Germany) was used for the compressive and flexural strength determination. The specimens were stored in a laboratory environment, during the polymerisation and subsequently until the time of testing. Compressive strength was tested from each formulation on three specimens and flexural strength on fractions of these beams, i.e., six specimens.

### 3.2. Cohesion with Concrete

The cohesion of epoxy composites was determined according to the EN 1542 standard [37]. The thickness of the layer of epoxy mortar applied on the surface of the concrete was approximately 5 mm, and the pull-off testing was performed seven days after the application of the epoxy composite using the DYNA pull-off tester PROCEQ Z16 (Proceq SA, Zürich, Switzerland). Three repetitions of the cohesion with concrete of each formulation were performed.

### 3.3. Dynamic Viscosity

Determination of the viscosity was performed using an MYR VR 3000 V1L rotational viscometer (MYR Viscotech, Ltd., El Vendrell, Spain). The temperature of the fresh mixtures was 20 °C at the start of the determination process and a spindle type R6 was used to measure the viscosity. The determination was performed immediately after the mixing of all epoxy composite components. Three repetitions of the dynamic viscosity determination of each epoxy mixture were performed.

### 3.4. Abrasion Resistance

The abrasion resistance was determined according to the EN 13892-3 standard [38] on three specimens from each type of epoxy composite with dimensions of 70 mm × 70 mm and a thickness of at least 30 mm, these being the same as the ones used in the hardness test. The test specimens were clamped in the N-1001 RT Böhm abrasion resistance tester (FORM + TEST Seidner & Co. GmbH, Riedlingen, Germany) on a grinding track on which the abrasive (20 g of corundum) was poured. Each test specimen was tested for 16 cycles, each of 22 rotations, with a sample load of 294 N. After each cycle, the sample was rotated 90° and a new abrasive was poured onto the grinding path. Abrasion resistance was expressed by reducing the volume after 16 cycles, in cm^3^ to 50 cm^2^.

### 3.5. Impact Resistance

The determination of impact resistance of the epoxy composites was performed according to the EN ISO 6272-1 standard [39] using falling weight onto a large striker area. Epoxy mortars were firstly applied in a thickness of approximately 4 mm to a cement particle board, then they were tested for impact resistance after seven days. Two samples from each formulation were tested and the test, at the same height of impact of the weight, was performed at 5 spots at least 2 cm apart.

### 3.6. Hardness

The surface hardness of epoxy composites was determined according to the standard EN ISO 868 [40]. A D type TQC hardness tester, LD0550 series was used to determine the hardness of materials based on epoxy resins (ER) showing high hardness. the hardness was determined on samples for determining the impact resistance at 5 spots at least 2 cm apart.

### 3.7. Thermal Expansion

The determination of the coefficient of linear thermal expansion (α) was performed using the CLASIC 30/100/15DIL dilatometer (CLASIC CZ, Ltd., Řevnice, Czech Republic,). The samples were of the same dimensions as in the determination of flexural strength (20 mm × 20 mm × 100 mm). The measurement was executed within a temperature range of 20–60 °C for approximately 18 h. Three repetitions were performed with each type of epoxy composite.

The coefficient of thermal expansion (*α*) was determined according to the following equation:

(1) $$\alpha =\frac{\Delta L}{L_{0}\times \Delta T}$$

where *α* is the coefficient of linear thermal expansion in (K^−1^), *L*_0_ is the original length of the specimen in (mm), Δ*T* is the temperature change in (K), and Δ*L* is the change in the length of the specimen *L* in (mm).

### 3.8. Effects of the Aggressive Environment

Firstly, the epoxy composites in a fresh state were applied to a thinner layer on an acetone-cleaned and dried laboratory slide. The specimens were left to polymerise for seven days on a clean underlay at a temperature of 20 ± 2 °C, Samples were then immersed into a lockable glass cuvette with a specific aggressive media, which in practice can affect epoxy composites primarily intended for the rehabilitation of concrete structures (40% H_2_SO_4_, 40% NaOH, 10% CH_3_COOH, gasoline, 10% NaCl, 30% H_2_O_2_). The chemically aggressive environment acted on the samples for 28 days at a temperature of 23 ± 2 °C. After this period, the samples were taken out of the aggressive medium, dried and then in accordance with Table 5, the effects of the aggressive environment on the epoxy composites were visually evaluated. This method was applied mainly in order to determine in which chemically aggressive environment the materials can be used and in which there is considerable degradation of the material. The evaluation system for the effects of the aggressive environment was prepared according to the expected behaviour of the epoxy composites in the aggressive media. The effect of the filler on the chemical resistance of epoxy materials was also monitored.

### 3.9. Microstructure—Digital Microscope and SEM

Microstructure changes were examined using a Keyence VHX950F digital optical microscope (Keyence Ltd., Osaka, Japan) due to the use of different filler types. Examination concentrated on looking for possible defects, air pores, microcracks, micro splits and micro blisters, also the means of distribution, the quality of homogenisation and eventual clustering of the filler within the cross-section of the specimen were evaluated. For monitoring the inner structure and microstructure, a TESCAN MIRA3 XMU (TESCAN Ltd., Brno, Czech Republic,) Scanning Electron Microscope (SEM), which allows the examination of materials with magnification up to 1,000,000×. Using the SEM, the samples of epoxy composites were examined at a resolution of up to 5000×. The acceleration voltage was 15 and 20 kV. Samples with a thickness of approximately 4 mm were prepared by sputtering by gold in a high vacuum.

### 3.10. FTIR

Using FTIR, it is possible to identify especially organic compounds that have a spectrum area within the wavenumbers of 400 to 4000 cm^−1^. Within the 4000–2500 cm^−1^ range, there is valence vibration of hydrogen, and a free binding of O-H absorbs at the highest wavenumbers at approximately 3600 cm^−1^. To determine the infrared spectrum, the Frontier PerkinElmer IR/NIR type spectroscope with an attenuated total reflection (ATR) diamond crystal as an adaptor was used. A small amount of the sample (10 mg) with a part size <100 µm homogenised with 300–400 mg KBr and a tablet was then extruded, which was then inserted into the FTIR spectrometer to determine its composition. Using FTIR, the spectrum of used treated hazardous waste (NS) and fly ash (FA) was also determined for comparison with individual spectrums.

### 3.11. CT Tomography

The computed tomograph (CT) Phoenix v|tome|x m 300 (Jess W Jackson & Assoc. Inc., Bristol, VA, USA) was used particularly to monitor the inner structure of epoxy composites, cohesion of polymer mortar with a concrete underlay, and the distribution of fillers in the epoxy matrix. This is a multi-purpose tomograph used for the analysis and 3D viewing of a wide spectrum of materials that operates with a voltage of 300 kV/500 W.

## 4. Results and Discussion

### 4.1. Compressive and Flexural Strength

**Figure 5:**
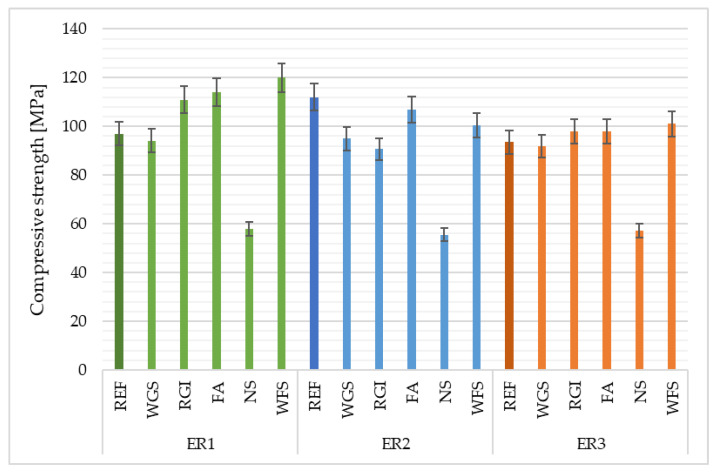
Results of compressive strength of epoxy composites depending on the type of filler and epoxy binder used.

**Figure 6:**
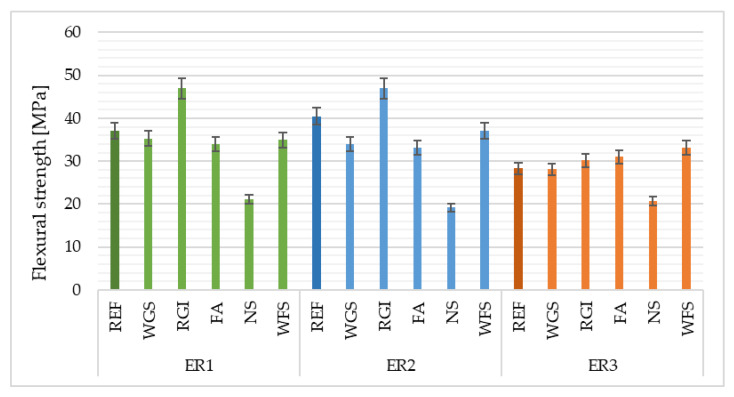
Flexural strength of epoxy composites depending on the type of filler and epoxy binder used.

From the obtained results of compressive (Figure 5) and flexural strength (Figure 6), it is obvious that the most significant reduction in strength occurs when neutralisation sludge (NS) is added (compared to the reference filler). This is primarily caused by the influence of discretely distributed individual grains. The highest strength was reached in the ER1 and ER3 samples when using WFS as a filler. The reference filler, in terms of compressive strength reached, works best with the ER2 resin; waste glass from solar panels QS solar reaches similar compressive strengths for all monitored binders; epoxy composites containing RGI filler reached their highest strength with the ER1 binder; NS behave the same with all binding resins; epoxy composite with WFS showed the highest strength when combined with ER1. The coarser-grained filler forms the skeleton of the epoxy composite, which is sufficiently strong, and, therefore, the samples containing WGS, WFS and REF showed the highest compressive strengths. According to the Technical Conditions for Rehabilitation of Concrete Structures III [41], repair materials for concrete with the R4 class static function must show a compressive strength of at least 45 MPa. This limit value was reached by all epoxy materials (Figure 5).

The highest flexural strength was reached in samples using recycled material from the production of glass insulation (RGI). This fact can be explained by the ‘rod-like shaped’ particle shape of RGI fine filler particles. Fly ash worked best with ER3 epoxy binder when almost the same flexural strength was achieved as in the case of a composite containing RGI filler. Neutralisation sludge (NS) showed comparable strengths in all used binders. Waste foundry sand (WFS) reached its highest strength when combined with the ER2 binder.

### 4.2. Cohesion with Concrete

**Figure 7:**
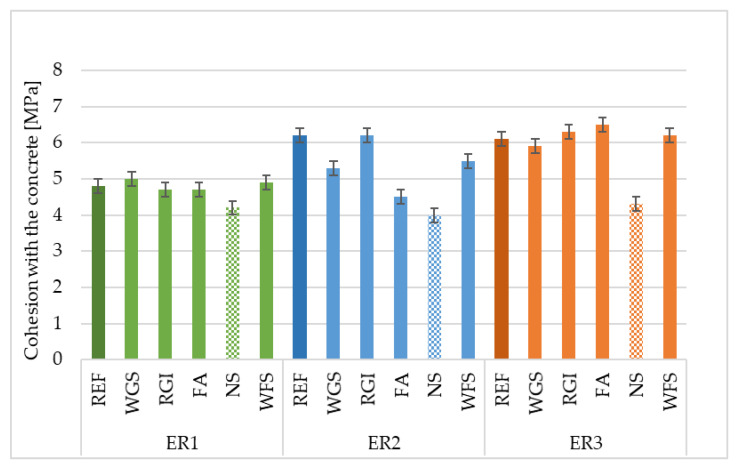
Cohesion of epoxy composites with concrete depending on the type of filler and epoxy binder used.

Failure in the sub-concrete layer occurred in all monitored samples, except for those containing NS. The highest cohesion with sub-concrete was reached by the reference filler in combination with the used ER2 binders: fly ash with ER3 and a waste foundry sand with ER3. The comparison of individual resins: WGS filler had the highest cohesion with sub-concrete combined with the ER3 epoxy resin. Recycled material from the production of glass insulation (RGI) reached the highest cohesion with the used ER2 and ER3 binders. Fly ash reached the highest cohesion with the ER3 binder. For samples using the NS filler, the value of cohesion with concrete was the lowest. In terms of the resin used, the highest cohesion with the sub-concrete with the waste foundry sand filler was reached by the ER3 binder. All examined samples met the requirements of the EN 1504-3 standard [42], which determines the minimum value of the cohesion with concrete 2.0 MPa for R4 class repair materials with a static function—see Figure 7. Courard et al. [43], stated that increasing substrate roughness promotes epoxy mortar adhesion due to better mechanical interlocking for high-strength concrete substrates. The pull-out strength of the epoxy adhesive systems that contained fillers based on micro silica improved up to 20% [44].

### 4.3. Dynamic Viscosity

**Figure 8:**
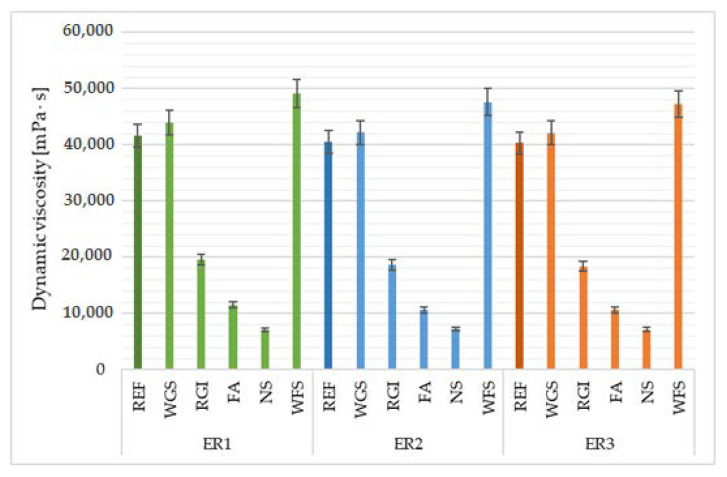
Dynamic viscosity of epoxy composites depending on the type of filler and epoxy binder used.

As is obvious from the results of the evaluation of dynamic viscosity (Figure 8), its values strongly depend on the type and particularly the amount of the filler, rather than on the type of binder used. Solid particles form a spatial network that highly increases resistance to flow. The lower the viscosity of the matrix (in higher temperatures) the greater its influence on the formed network; low viscosity fluid is able to destroy the formed network and, in this way, lower the viscosity [45]. The lowest values of dynamic viscosity were reached for materials using the neutralization sludge, the second-lowest dynamic viscosity was recorded in samples with fly ash, followed by the recycled material from the production of glass insulation. The highest values were reached for the waste foundry sand (WFS), and slightly lower values in samples with WGS and with the reference filler. The lowest values of the dynamic viscosity were reached by the mixture containing neutralization sludge (NS), which was caused by the highest value of the specific surface area for this type of filler since a greater amount of epoxy resin, which is viscose, is used for coating the individual particles.

### 4.4. Abrasion Resistance

**Figure 9:**
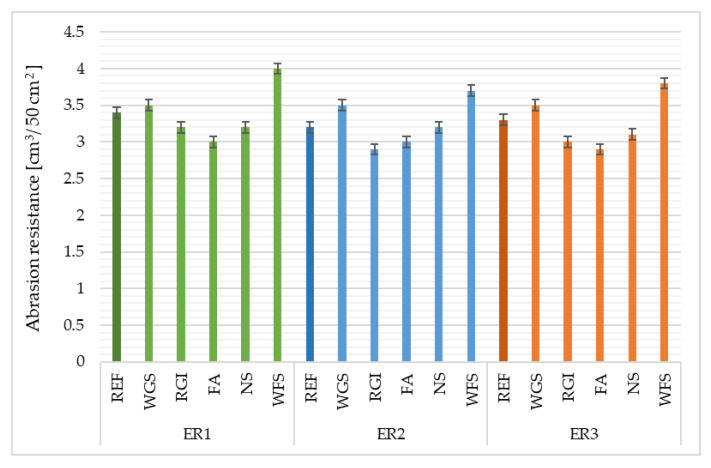
Abrasion resistance of epoxy composites depending on the type of filler and epoxy binder used.

The highest abrasion resistance was observed in samples using fly ash (FA) as a filler and the ER1 as a binder, alternatively, the lowest ability to resist the abrasion with the highest volume reduction was observed in composites containing waste foundry sand (WFS) as a filler and in all types of binders. Based on the comparison of the abrasion resistance results (Figure 9) it is possible to state that coarser fillers of regular, spheric, monoclinic to tetragonal shape (REF, WFS), but also irregular shape (WGS), have a negative influence on the abrasion resistance of epoxy composites. This is caused by easier abrasion of the grains by corundum sand than in composites with finer fillers (FA, RGI, NS), where the contact zone between the filler and the binder is greater in total volume, and particularly on the surface, the fillers are better coated by the polymeric matrix. The wear resistance of polymers is improved by the addition of fillers [46,47]. Yousif et al. [48] found out that the epoxy composite experiences high wear resistance when it is subjected to fine sand particles followed by grain and finally coarse sand.

### 4.5. Impact Resistance

**Figure 10:**
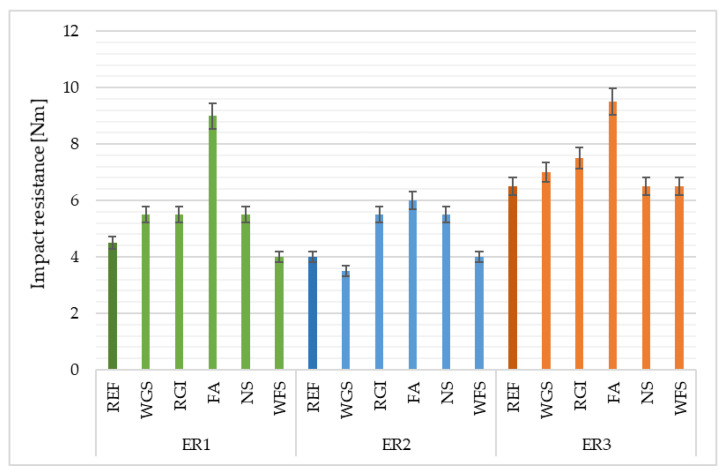
Impact resistance of epoxy composites depending on the type of filler and epoxy binder used.

The highest impact resistance was recorded in the epoxy composites containing fly ash as the filler, whilst the highest values were observed with the samples based on ER1 and ER3 resins—see Figure 10. Generally, it is possible to assess that the materials containing finer fillers (FA, RGI) showed better impact resistance. Materials with more coarse fillers were more fragile and, therefore, demonstrated a lower impact resistance.

### 4.6. Hardness

**Figure 11:**
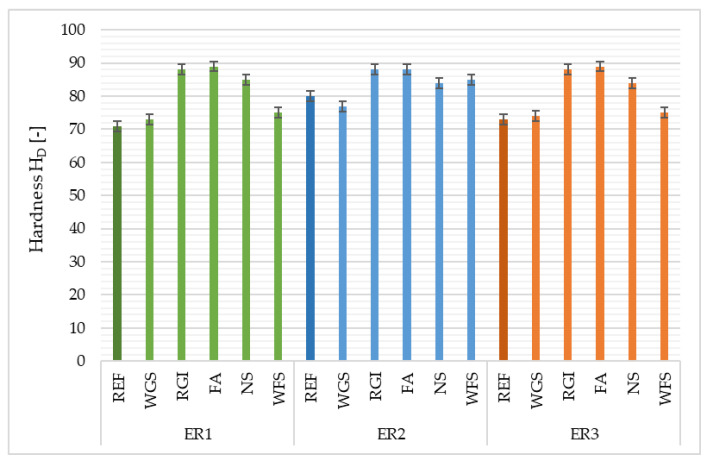
Surface hardness of epoxy composites depending on the type of filler and epoxy binder used.

Shore D hardness was tested on samples aged 28 days and the results are shown in Figure 11. The hardness values depend on the time of the action of a foreign body, on its geometry and material properties, load weight, elastic properties of the tested materials and on the temperature during the test. The hardness of polymers is subject to more complex regularities than materials of a metallic nature, as demonstrated by polymer properties such as relatively low elasticity and viscoelastic behaviour. Zhang et al. [31] reported that low crosslink density decreases the hardness of polymer composites. Epoxy materials with smaller particles (d < 208 µm) have a homogeneous microstructure, and a volume fraction of the particulate waste greater than 300 µm can be used to obtain any useful increase in hardness. This produced a hardness gradient and a hardened surface five times harder than the bare resin matrix obtained [33].

It was verified that the influence of the type and amount of filler can slightly influence the results for material hardness. Furthermore, it was proven that materials containing finer particles as a filler (FA, RGI) show higher values of the Shore D hardness than the materials filled by WGS, NS and WFS, in which the particle size ranged up to 1.5 mm. The highest value of Shore D hardness was recorded for samples using the fly ash filler. Moreover, it is possible to see that the type of resin used did not have any influence on the hardness of the composite surface. On the contrary, the lowest values were observed in samples using the reference filler and WGS.

### 4.7. Coefficient of Linear Thermal Expansion

The coefficient of linear thermal expansion (α) is a particularly important parameter of polymers used mainly in engineering applications. A low-value α is often desirable for acquiring dimension stability and this can be achieved by the addition of a solid and fine graphite filler. It was observed that the α value of the hardened epoxy resin (ER) is 60 × 10^−6^ K^−1^ and by the adding of 2.5% (weight) of graphite plates, it reduces to 36–41 × 10^−6^ K^−1^, which is approximately 30–40% lower in value [49]. The main cause of the decrease in the α value, in this case, is considered to be the subtle dispersion and rigidity of graphite plates in the ER matrix, which can inhibit the expansion of polymeric chains during the rise in temperature.

**Figure 12:**
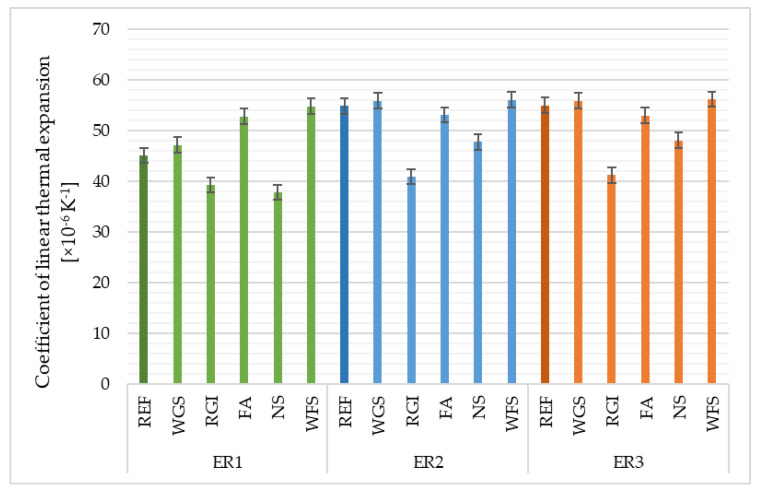
Coefficient of linear thermal expansion of epoxy composites depending on the type of filler and epoxy binder used.

The crosslinking point mutually pulls molecular chains under a micro-Brownian motion, thereby preventing the molecular chains from expanding with rising temperature. In the rubbery region (190–250 °C), the coefficient decreases as the crosslinking density of cured resin and on the contrary increases in the glassy region (50–140 °C), when an increase in the crosslinking density occurs [50]. From the results shown in Figure 12, it is obvious, as stated by Wong [51], that the coefficient of the linear thermal expansion decreases with a decrease in the filler content (NS, RGI). The linear thermal expansion was higher with waste foundry sand, fly ash, silica sand and waste glass from solar panels, which all build a stronger skeleton than finer fillers. The type of resin used had a negligible effect on the α value.

### 4.8. Effects of the Aggressive Environment

Organic acids, such as HCOOH, CH_3_COOH and CH_3_CH_2_COOH, are quite weak acids and so they are much less dissociated. These acids mainly act as solvents; their effect leads to the creation of surface blisters and the separation of segments of macromolecular chains. It was also proven that an increase in chemical resistance can be achieved by the implementation of fillers that are able to react with a diffusing acid. Inner fillers or pigments (TiO_2_, graphite, soot, chromium oxides), present in the epoxy matrix, increase the diffusion of an aggressive (corrosive) medium since the penetration at the pigment/binder interface occurs along the pigment particles [52].

**Figure 13:**
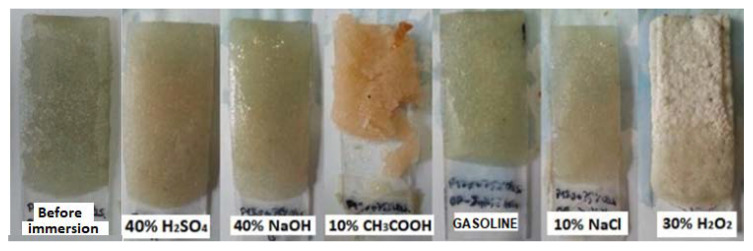
Samples of epoxy composites composed of the waste glass (WGS) and the ER1 polymeric matrix after 28 days of exposure to various chemically aggressive environments.

**Figure 14:**
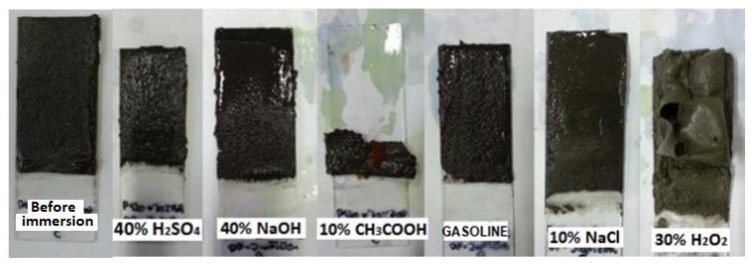
Samples of epoxy composites composed of the fly ash (FA) and the ER2 polymeric matrix after 28 days of exposure to various chemically aggressive environments.

**Figure 15:**
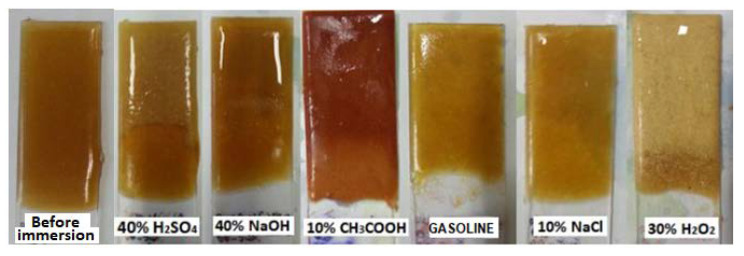
Samples of epoxy composites composed of the RGI filler and the ER3 polymeric matrix after 28 days of exposure to various chemically aggressive environments.

Based on the evaluation of the executed accelerated chemical resistance test in Table 6, it is obvious, from Figure 13, Figure 14 and Figure 15, that no damage to the structure of the epoxy composites or their surface occurred due to exposure to NaOH, NaCl or distilled water. Regarding the methods using liquid-phase decomposition, supercritical or subcritical fluid decomposition and peracid decomposition have been widely investigated [53,54,55,56,57]. From the results of the test, it is obvious that epoxy resins showed poor resistance to acetic acid solutions and hydrogen peroxide. Oxidative degradation occurs because of exposure to H_2_O_2_ [58]. The degree of damage observed was similar for all types of resin used. Only the ER3-based epoxy composites achieved slightly better chemical resistances, particularly when exposed to CH_3_COOH. The higher chemical resistance of the ER3 resin is related to the fact that it contains formaldehyde and phenol in the A component, thanks to which it is possible to rank it among Novolac resins. The added functionality of the phenolic resin increases the ability of the resin to crosslink, creating a stronger polymer network with high resistivities. The high chemical and solvent resistivities and temperature compatibility of epoxy phenolic resins are most useful when used in high-performance applications and in corrosion resistance [59,60]. Samples with NS and the ER3 resin also exhibited high chemical resistance, and they can be used in a chemically aggressive environment to avoid a possible release of contaminants from the material to the environment, thus, ensuring appropriate environmental protection.

### 4.9. Microstructure—Digital Microscope and SEM

**Figure 16:**
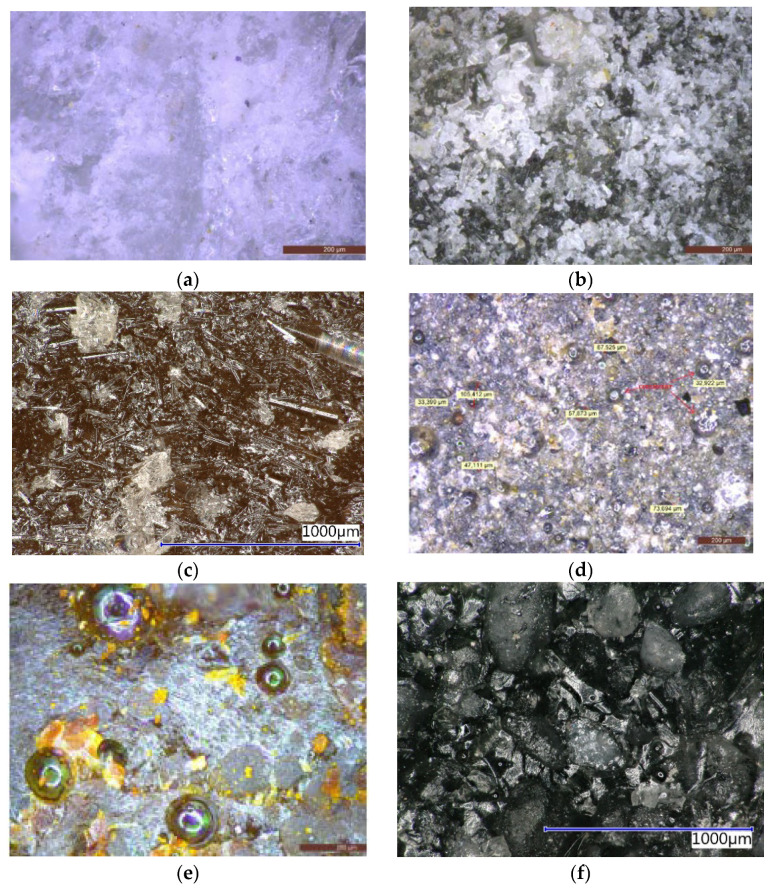
Detail of the structure of prepared epoxy composites with different filler using the digital microscope: (**a**) REF—Silica sand 100×; (**b**) WGS—Waste glass from solar panels QS Solar 100×; (**c**) RGI—Recycle material from the production of glass insulation 200×; (**d**) FA—Fly ash 200×; (**e**) NS—Neutralisation sludge 100×; (**f**) WFS—Waste foundry sand 200×.

From the optical digital microscope images (Figure 16), it is possible to see how the filler is incorporated into the polymeric matrix. All types of fillers used are perfectly coated with the epoxy resin. Filler particles are equally distributed in the mixture and air pores have shown a maximum diameter of 100 µm.

**Figure 17:**
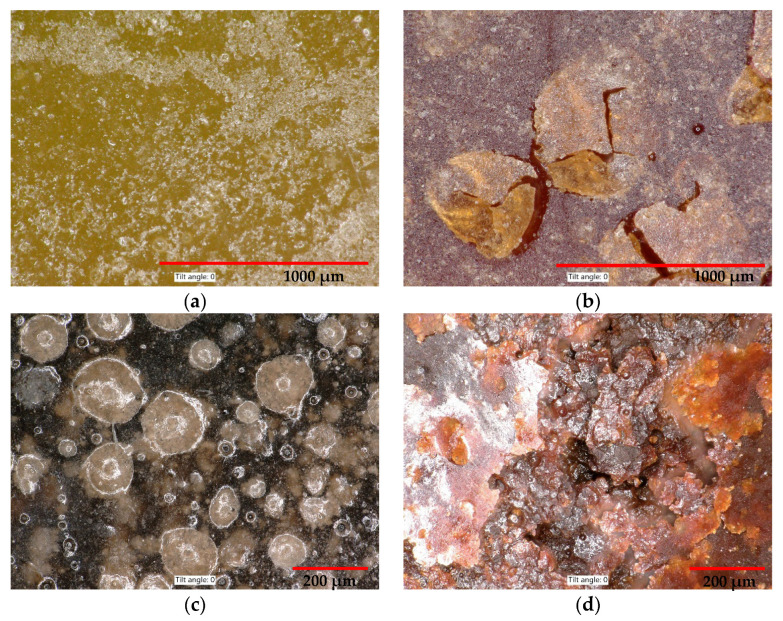
Samples of the epoxy composite with REF and NS filler following exposure to chemical stress: (**a**) REF in H_2_SO_4_, magn. 200×; (**b**) REF in CH_3_COOH, magn. 200×; (**c**) NS in H_2_SO_4_, magn. 100× (**d**) NS in CH_3_COOH, magn. 100×.

The samples were also microscopically examined (Figure 17) following exposure to various chemically aggressive environments. In comparison with the reference images, it is obvious that after the exposure to a solution of sulphuric acid, no surface damage to the polymeric composite occurred. Acetic acid had a significant degrading influence on the samples—in Figure 17b,d, there are evident cracks and peeling of the epoxy thermoset layers from the surface.

**Figure 18:**
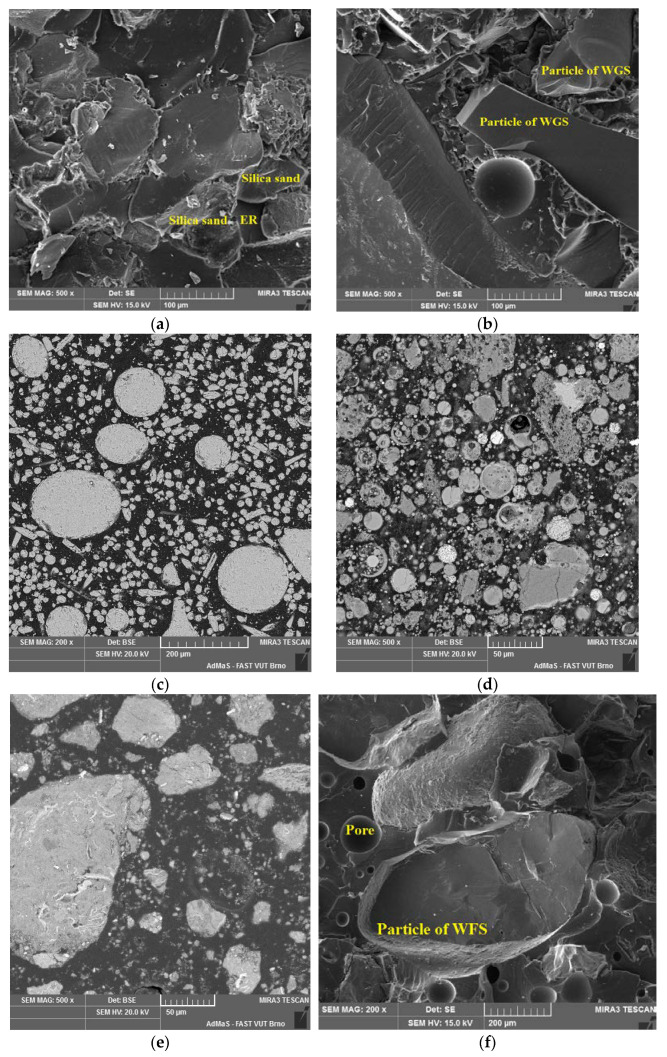
SEM photomicrographs of the epoxy composites with different fillers: (**a**) REF—Silica sand, 500×; (**b**) WGS—Waste glass from solar panels QS Solar 500×; (**c**) RGI—Recycle from the production of glass insulation 200×; (**d**) FA—Fly ash 500×; (**e**) NS—Neutralization sludge 500×; (**f**) WFS—Waste foundry sand.

From the photomicrographs obtained from SEM (Figure 18), it is obvious that the filler particles are perfectly coated with the polymer matrix in all epoxy materials. Similar results were recorded in all epoxy binders. No chemical bond between epoxy matrix and filler particles occurred in any binder (ER1, ER2 and ER3). The different parts of a filler are only physically bound together in the epoxy resin. No evident clusters of particles are present, and they do not occur in the area with an increased number of air pores either. In Figure 18d, there are clearly visible cenospheres that commonly occur in the high-temperature fly ash. They are equally distributed in the sample and no clustering of these particles has occurred. From the image of the neutralization sludge sample (Figure 18e), it is obvious that particles of this filler are perfectly incorporated into the epoxy matrix and, therefore, there is no danger that any negative release or leaching of pollutants into the environment can occur. From the photomicrographs, it is not apparent if any chemical reactions between the binder and filler were present, and no new structures were observed.

**Figure 19:**
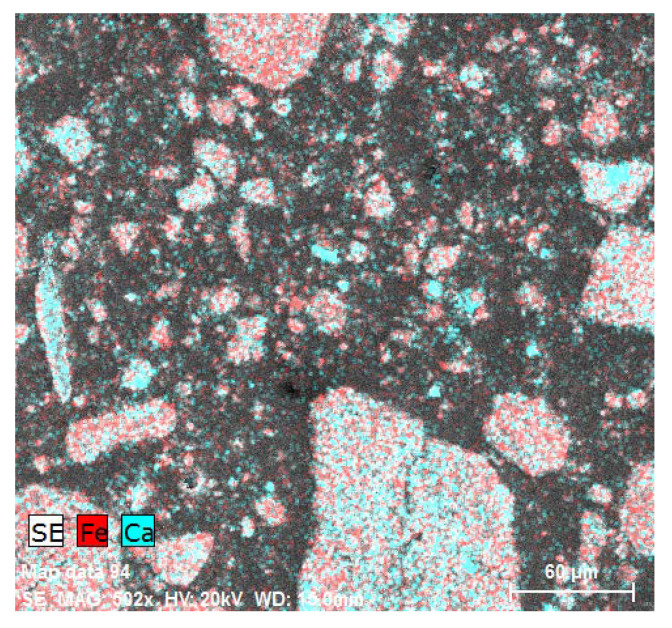
The EDX analysis of NS particles in the polymeric matrix—graphic illustration of the elements present.

From Figure 19, the chemical composition of parts of the epoxide composite specified by the EDX analysis is evident. Blue areas represent calcium and red areas represent iron. From the photomicrograph supported by the EDX evaluation, it is clear that contaminants from particles of neutralisation sludge (NS) have not been released into the polymeric matrix, thus ensuring its perfect incorporation into the inner structure is ensured.

### 4.10. FTIR

**Figure 20:**
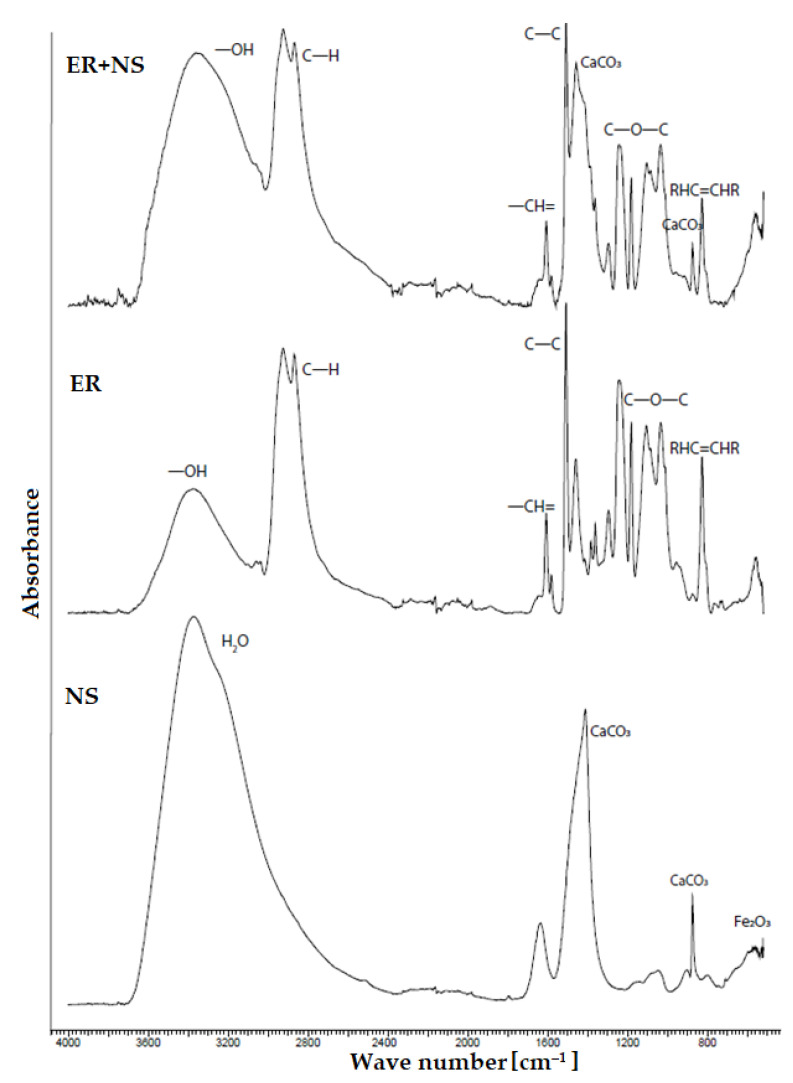
FTIR analysis—comparison of spectrums of neutralisation sludge (NS) with epoxy resin, pure epoxy resin (ER) and NS.

**Figure 21:**
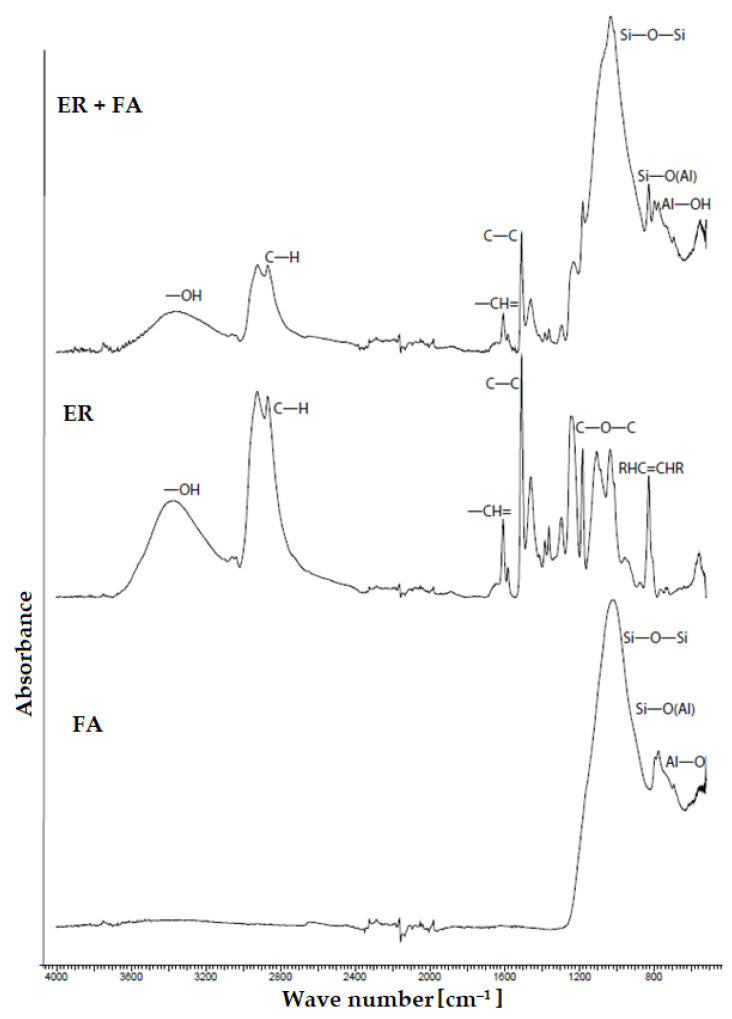
FTIR analysis—comparison of spectrums of fly ash (FA) with epoxy resin, pure epoxy resin (ER) and FA.

Due to the incorporation of hazardous waste (NS) into the polymeric matrix (Figure 20), no new chemical bonds between the binder and filler have occurred. In the evaluation of the spectrum itself, in the material with fly ash (Figure 21), aluminosilicate strips and silicates from the fly ash itself were found, whilst the bonding of the -OH group to the aluminous element of the fly ash occurred, which was possible to observe at a wavenumber of 850 cm^−1^. In the sample containing NS filler, carbonates stripes (CaCO_3_) were observed and at the start of the middle area of IR, iron oxides were most likely identified, which were also observed in the chemical analysis of the sludge itself. In the organic component, only compounds typical for epoxides were observed, such as the oxirane ring (C-O-C), C-H bonds and other aromatic compounds, and these findings were observed in all ER used. In all spectrums of the epoxy composites, the hydroxyl (-OH) group with a value of approximately 3400 cm^−1^ was detected.

### 4.11. CT Tomography

**Figure 22:**
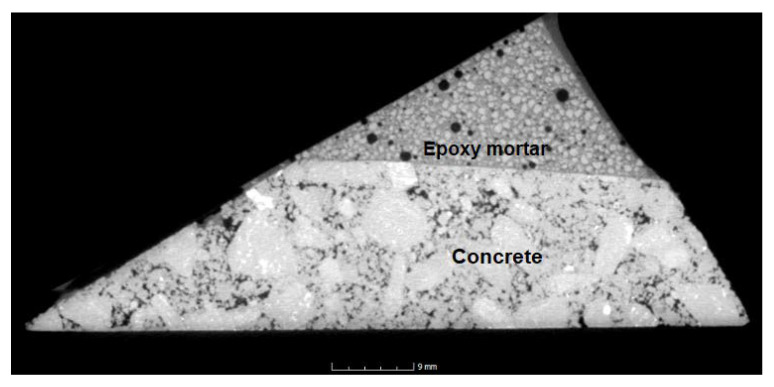
CT scan of the corner reprofiled by the reference epoxy composite.

**Figure 23:**
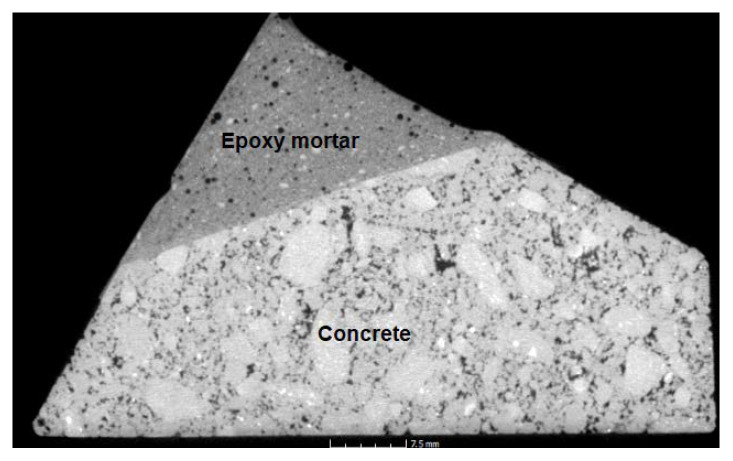
CT scan of the corner reprofiled by the epoxy composite containing RGI.

**Figure 24:**
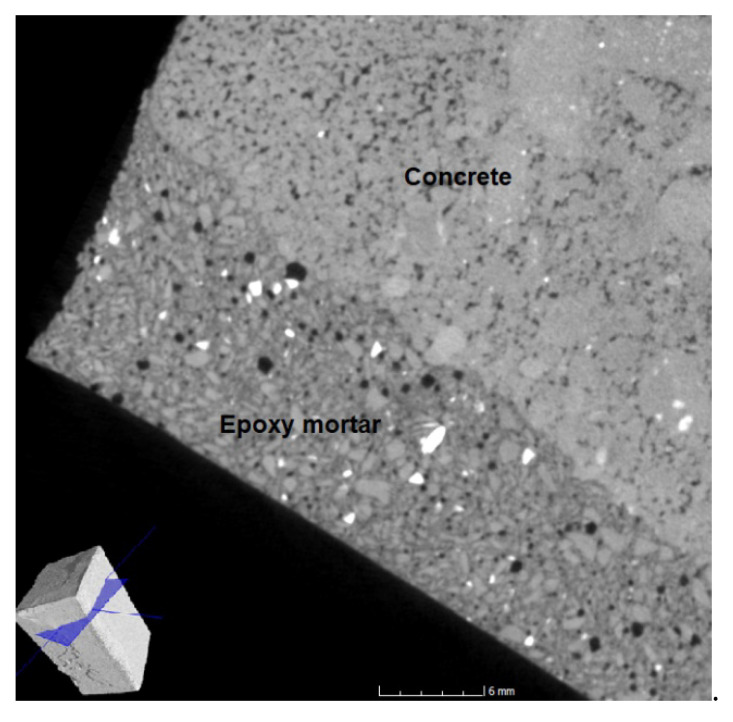
CT scan of the corner reprofiled by the epoxy composite containing the waste glass with the location indicating the scan in the sample.

**Figure 25:**
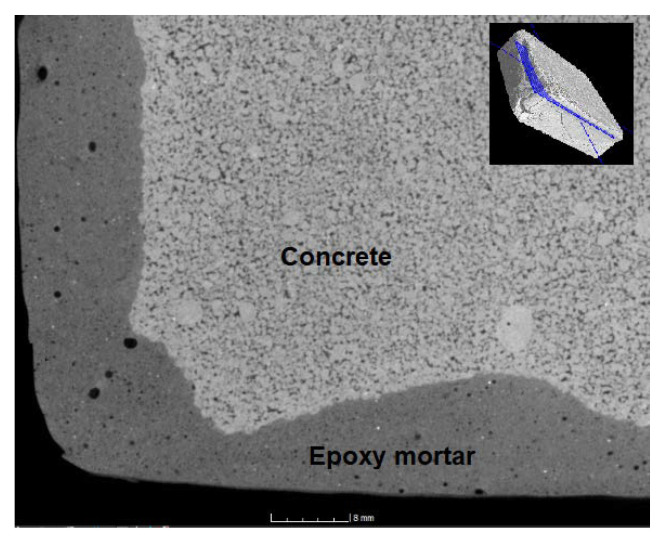
CT scan of the corner reprofiled by the epoxy composite containing the fly ash with the location indicating of the scan in the sample—perpendicular plane of the cut.

In the CT scans (Figure 22, Figure 23, Figure 24 and Figure 25), the interface of the connection of the epoxy composite on a concrete underlay (concrete curb with damaged corner) can be clearly seen. From the images, it is obvious that a perfect connection of the epoxy mortar to the underlay concrete has occurred, while no separation layer has been formed. Edges are smooth without any evident defects; the only defects are caused by the provisory laboratory formwork. The formwork used in practice is more suitable. Moreover, it is also evident from the images that a perfect distribution of the filler’s components has occurred. In Figure 22, a minor defect can be seen caused by the lower viscosity of the mixture and the segregation of the binding component due to gravitational power and differing densities of both components. However, this visual defect has no impact on the resulting physical and mechanical parameters or the long-term durability. From all CT images, it is evident that the epoxy composites contained at least a minimum number of open pores.

In Figure 25, lighter grains of fly ash, which are equally dispersed in the epoxy matrix, can be seen. The black areas are air pores that are equally distributed throughout the composite. Even in this CT scan, the perfect cohesion of the repair material to the concrete underlay can be seen. The colour difference is caused by the different density of the concrete (2400 kg/m^3^) and epoxy composites (1500 kg/m^3^), fly ash has a density of approximately 2600 kg/m^3^, and, therefore, its grains can be clearly seen in the mixture. From the SEM analysis and the CT tomography, it is obvious that finer and coarser particles of fillers are dispersed equally throughout the composite.

## 5. Conclusions

It was determined that the type of filler used has a more significant influence on the resulting properties of the mixture than the type of resin itself. Fillers that were used for the experiment were mainly secondary raw materials, which were chosen to have as many different shapes and particle sizes as possible. Regarding the obtained results, it can be stated that coarser grains of a filler with a more equal geometric shape (REF, WFS) have a positive influence especially on the compressive strength thanks to less compression of the filler grains in the epoxy composite. Alternatively, epoxy composites containing finer fillers (FA, RGI) show higher flexural strength, better abrasion resistance and better impact resistance. Epoxy mortars containing quartz sand as a filler were also tested in order to compare the results of tested materials with the available materials containing only primary row materials. Compared to other commercial epoxy materials, even better mechanical properties have been achieved with epoxy composites containing waste materials as fillers. The highest flexural strength was recorded in samples containing the RGI filler, which was the most heterogeneous in terms of particle shape, and it can be assumed that the ‘rod-shaped’ particles of a filler had a positive influence on the load resistance. Based on the evaluation of the FTIR analysis, no new chemical bonds between the filler and binder particles were observed. The highest strength was recorded in the ER1 and ER3 samples using the waste foundry sand filler. No damage to the polymer structure occurred due to exposure to NaOH, NaCl or distilled water. The best resistance against the chemically aggressive environment was observed in the ER3 Novolac epoxy resin, although composites based on ER1 and ER2 also showed outstanding chemical resistance. The filler type has no particular influence on the chemical resistance. The filler components were only physically bonded to the epoxy matrix. It was also proven that it is possible to incorporate hazardous waste (NS) into the epoxy matrix. Newly developed epoxy materials with a high content of by-products can be used in practice (building applications) due to their high mechanical parameters and chemical resistance, e.g., as polymer mortars, rehabilitation materials, polymer concretes, adhesives and grouts.

## Figures and Tables

**Table 1:** Polymer binder types used—basic bases, hardener type used and mixing ratio.

Epoxy Binder	Component A	Component B	Mix Ratio by Weight of Resin to Hardener
ER 1	Epoxy resin, (alkoxymethyl) oxirane (C12–C14 alkyl), solvent naphtha (petroleum), light aromatic	Benzyl alcohol, polymer with benzenamine, hydrogenated formaldehyde, 2,4,6-tris (dimethylaminomethyl) phenol, 1,4-bis(aminocyclohexyl)methane	3.2:1
ER 2	Epoxy resin, (alkoxymethyl) oxirane (C12–C14 alkyl), solvent naphtha (petroleum), light aromatic	Benzyl alcohol, fatty acids, reaction products with triethylenetetramine, 3-(aminomethyl)-3,5,5-trimethylcyclohexan-1-amin, bisfenol Am-fenylenbis (methylamin), N,N-dimethylpropan-1,3-diamin, 2,4,6-tris(dimethylaminomethyl) phenol	1.8:1
ER 3	Epoxy resin, oxirane, formaldehyde,oligomeric reaction products with 1-chlor-2, 3-epoxypropane and phenol	Trisphenol, benzyl alcohol, Methylene oxide polymer with benzenamine, hydrogenated, 4, 4-Methylenebis	1.8:1

**Table 2:** Properties of epoxy binders used within the research.

Epoxy Binder	Specific Weight at 20 °C (kg/L)	Pot Life at 20 °C (min)	Max. Moisture of Substrate (%)	Dynamic Viscosity (mPa·s)
ER 1	1.30	20	5	2200
ER 2	1.11	2	12	2160
ER 3	1.10	20	4	2150

**Table 3:** Chemical composition of the fillers (% dry matter).

Parameter	REF	WGS	RGI	FA	NS	WFS
SiO_2_	99.5	71.0	50.6	53.4	1.12	94.0
Al_2_O_3_	0.41	0.499	13.5	18.34	0.53	1.72
Fe_2_O_3_	0.028	0.11	0.33	10.57	56.6	0.387
Na_2_O	0.029	12.4	0.35	0.21	0.08	1.99
K_2_O	0.222	0.171	0.58	0.91	0.06	0.726
CaO	0.038	8.45	21.2	4.13	15.5	0.174
MgO	-	4.04	0.45	0.89	0.05	0.057
MnO	-	0.006	-	-	-	0.011
ZnO	0.01	-	-	-	-	-
SrO	-	0.005	0.11	-	-	-
CuO	-	-	-	-	0.326	-
NiO	-	-	-	-	0.014	-
Cr_2_O_3_	<0.004	0.005	-	-	0.023	0.081
TiO_2_	0.031	0.023	0.32	-	-	0.037
SO_4_	-	-	-	0.25	0.47	-
PbO	0.02	-	-	-	0.262	-
V_2_O_5_	-	-	-	-	1.43 × 10^−4^	-
TOC	-	-	-	5.2	0.02	-

REF—Reference filler, WGS—Waste glass from solar panels, RGI—Waste from the production of mineral insulation boards, FA—Fly ash, NS—Neutralisation sludge, WFS—Waste foundry sand

**Table 4:** Basic properties of the fillers.

Parameter	REF	WGS	RGI	FA	NS	WFS
Specific gravity (kg/m^3^)	2662	2509	2620	2390	2960	2680
Specific surface area (cm^2^/g)	330	390	900	3410	7800	430

**Table 5:** Evaluation system for accelerated chemical resistance test designed with regard to the expected behaviour of the epoxy composites in the aggressive media.

Indication of a Breach	Evaluation Criterion
7	The material shows no changes
6	Colour changes
5	Swelling + colour changes
4	Peeling the material off the slide
3	Peeling the material off the slide + swelling + colour changes
2	Peeling the material off the slide + softening
1	Complete decomposition of the material

**Table 6:** Evaluation of the chemical resistance.

Material	Aggressive Medium	Concentration	REF	WGS	RGI	FA	NS	WFS
ER1	H_2_SO_4_	40%	7	7	6	7	6	7
ER2			7	7	6	7	6	7
ER3			7	7	7	7	6	7
ER1	NaOH	40%	7	7	7	7	7	7
ER2			7	7	7	7	6	7
ER3			7	7	7	7	7	7
ER1	CH_3_COOH	10%	1	3	2	1	1	2
ER2			1	3	2	1	1	2
ER3			2	3	3	2	2	2
ER1	Gasoline	-	7	7	7	7	7	7
ER2			7	7	7	7	6	7
ER3			7	7	7	7	7	7
ER1	NaCl	10%	7	7	7	7	7	7
ER2			7	7	7	7	7	7
ER3			7	7	7	7	7	7
ER1	H_2_O_2_	30%	5	6	6	2	3	5
ER2			5	6	5	2	4	5
ER3			5	6	6	3	4	6
ER1	Distilled water	-	7	7	7	7	7	7
ER2			7	7	7	7	7	7
ER3			7	7	7	7	7	7

## Data Availability

All the data is available within the manuscript.
